# From theory to practice: Revealing the real-world impact of cognitive behavioral therapy in psychological disorders through a dynamic bibliometric and survey study

**DOI:** 10.1016/j.heliyon.2024.e37763

**Published:** 2024-09-14

**Authors:** Hadi Dhafer Hassan Kariri, Abdulmohsen Almubaddel

**Affiliations:** a Department of Psychological and Behavioral Science, College of Arts and Humanities, Jazan University, Jazan, Saudi Arabia; b Department of Psychology, King Saud University, Riyadh, Saudi Arabia

**Keywords:** *Bibliometrics*, *Trending topics*, *Cognitive behavior therapy*, *Mobile health*, *Performance*, *CBT in Saudi Arabia*

## Abstract

**Background:**

Cognitive behavior therapy (CBT) is a proven treatment for many psychological disorders. It has been extensively studied and is effective for anxiety, depression, and schizophrenia. However, a bibliometric analysis of the CBT literature for these disorders is needed. This study reviewed this field's research and identifies key trends, influential studies, and gaps.

**Methods:**

Using MeSH-generated keywords and PRISMA guidelines, the Scopus database retrieved bibliographic data without temporal or geographical constraints. Data-driven studies were analyzed for performance, collaborative pattern, impact, co-word frequency, knowledge structure, and trending topics using R-package-based Bibliometrix and VOSviewer applications. The current study applied bibliometric and statistical analyses.

**Results:**

Scopus yielded 2757 studies since inception in 1979. The polynomial regression coefficient of 0.945 (R^2^) indicates a strong positive trend, and the research has increased at an annual rate of 12.67 %. Scholars from five countries had a noteworthy production of CBT research in treating PsD, namely the United States, the United Kingdom, Germany, Australia, and the Netherlands. Depression, oppositional defiant disorder, schizophrenia, and implementation are the clusters in the CBT research map. The thematic map needed to meet the desired criteria for representing all anticipated themes. Thematic evolution is evidence of noticeable changes that contributed to the creation of new research clusters and the disappearance of some of them. COVID-19 has significantly impacted the adoption and efficacy of internet-based interventions for mental health. The cross-sectional study provides valuable insights into the development and dissemination of CBT in Saudi Arabia, emphasizing the importance of training, awareness, and research.

**Conclusions:**

This study proposes that further investigation be conducted in contemporary literature to create a comprehensive framework for enhancing policy decisions regarding CBT.

## 1 Introduction

Cognitive-behavioral therapy (CBT) is a psychotherapeutic approach that identifies and modifies maladaptive cognitive and behavioral processes [1]. CBT assumes that our cognitive processes, emotional experiences, and behavioral patterns are interconnected and can influence each other [2]. A trained professional helps patients become aware of their negative or distorted cognitive patterns and beliefs in CBT [3]. Cognitive patterns and belief systems may cause emotional issues and maladaptive behavior. Recognizing and questioning these harmful patterns allows people to develop more pragmatic and flexible cognitive and behavioral approaches [1,4].

Research on CBT has helped us understand this therapy and its effectiveness in treating various mental health disorders. CBT is widely recognized and empirically supported as a therapeutic intervention due to the many studies that have examined its efficacy, processes, and suitability. Numerous efficacy studies have shown that CBT reduces symptoms and improves outcomes for many disorders. For instance, empirical research has shown that this intervention treats generalized anxiety disorder, social anxiety disorder, and panic disorder. CBT can treat depression, PTSD, OCD, eating disorders, substance abuse, and many other mental health conditions [[5], [6], [7], [8]].

Research emphasizes the importance of understanding how CBT provides therapeutic results. Cognitive restructuring—identifying and examining negative or distorted cognitive patterns and beliefs—is central to CBT. This process helps people develop pragmatic and adaptable cognitive frameworks. Exposure and response prevention help people face and overcome avoidance and fear. These mechanisms and other factors make CBT effective [1,2,9]. CBT's long-term benefits have been studied extensively. CBT improves people long after the intervention, according to subsequent studies. This shows CBT's long-term impact and ability to change lives [1,2,9]. Additionally, CBT now includes diverse populations and settings. Many studies have examined CBT's efficacy in children, adolescents, adults, and older adults [10]. These studies tailor interventions to each age group's needs and traits. CBT is used in individual, group, and self-help settings [2,3]. Scholarly studies have also examined the incorporation of CBT into online programs and mobile apps to improve accessibility and expand services to those who cannot access in-person therapy [11,12].

CBT has been compared to other treatments in comparative effectiveness research [1,13]. CBT outperforms medication and psychodynamic therapy for many mental health disorders [13]. These previous findings demonstrate the value of CBT in psychotherapy. CBT also emphasizes therapist competence and protocol adherence. Studies show that therapist proficiency is positively correlated with treatment outcomes, emphasizing the importance of training and supervision for cognitive-behavioral therapists [[14], [15], [16]].

CBT has improved our understanding of this therapeutic modality. Effectiveness studies, mechanisms of change studies, long-term benefits analyses, and diverse populations and settings have established CBT as a scientifically supported treatment for a variety of mental health disorders. Further research in this area will improve our understanding and implementation of CBT, improving outcomes for those who need psychological help. A bibliometric review of CBT research provides a quantitative overview of its progress, contributions, and impact. It discusses influential authors, institutions, collaborative networks, and CBT research trends [17]. Bibliometric analysis helps researchers, clinicians, and policymakers understand CBT and make informed decisions about future research, resource allocation, and mental health evidence-based practice. The goal of this study is to conduct a comprehensive bibliometric investigation on the data-driven output of CBT research. Additionally, the study aims to explore the opinions and experiences of practitioners in Saudi Arabia who utilize CBT. By examining the research landscape and gathering insights from practitioners, this study seeks to enhance our understanding of the effectiveness and utilization of CBT in the context of psychological disorders in Saudi Arabia.

## 2 Materials and methods

### 2.1 Database selection

To extract bibliographic data on CBT and its use in treating mental illnesses from Scopus, the following method was employed. Scopus was chosen as the preferred database for this study due to its extensive coverage of scientific literature across multiple disciplines, including psychology and psychiatry. Scopus provides a comprehensive collection of scholarly articles, conference papers, and reviews [18], making it suitable for retrieving relevant CBT research articles.

### 2.2 Search strategy

**Fig. 1:**
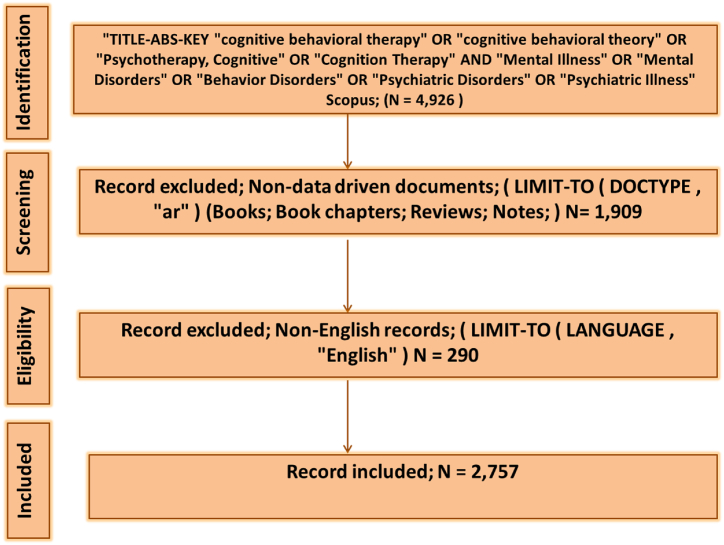
Data extraction followed the PRISMA (Preferred Reporting Items for Systematic Reviews and Meta-Analyses) guidelines [20]. This Figure is related to the bibliometric study.

The search query was constructed using the keywords ("cognitive behavioral therapy" OR "cognitive behavioral theory" OR "Psychotherapy, Cognitive" OR "Cognition Therapy") AND ("Mental Illness" OR "Mental Disorders" OR "Behavior Disorders" OR "Psychiatric Disorders" OR "Psychiatric Illness"). These keywords were derived from the MeSH database [19] using the provided links (http://id.nlm.nih.gov/mesh/D001523 and http://id.nlm.nih.gov/mesh/D015928). Data extraction followed the PRISMA (Preferred Reporting Items for Systematic Reviews and Meta-Analyses) guidelines [20] as shown in Fig. 1.

### 2.3 Eligibility and data extraction

No time or geographical limits were set to ensure a comprehensive collection of relevant articles. However, research articles published in languages other than English were excluded to maintain consistency and to focus on English-language literature. The top-cited articles were screened based on their titles and abstracts. Data were extracted from the selected articles, including publication year, authors, journal, citations, keywords, outcomes, and any other relevant information. Data was extracted using BibTex and CVS formats.

### 2.4 Data analysis

VOSviewer and Bibliometrix are well-known applications for assessing bibliographic data and conducting bibliometric analyses [21,22]. VOSviewer is a program that allows for the visual exploration and analysis of bibliographic networks. It assists researchers in identifying patterns, associations, and concentrations within a collection of publications. VOSviewer can generate co-authorship networks. Researchers can gain insight into collaboration patterns and knowledge domains by displaying bibliographic data visually [21,23]. In VOSviewer, Total Strength Links (TLS) measures the cumulative strength of bibliometric network connections for a specific author or publication. Based on its network connections, it shows the item's prominence. Bibliometrix, on the other hand, is a R package designed specifically for bibliometric analysis [22]. It provides a variety of statistical functions and methodologies for analyzing and interpreting bibliographic data. Utilizing Bibliometrix, researchers can calculate a variety of bibliometric indicators, including publication counts, and citation counts. It also provides mapping and segmentation tools for research publications based on keywords, authors, and journals [22]. The H-index is a bibliometric measure that quantifies the impact of a researcher's work based on the number of publications and the number of citations those publications have received. It represents the highest number of papers (h) that have received at least h citations each. The G-index ranks researchers based on the distribution of their citations. It calculates the highest number of highly cited papers that received the same number of citations as the total number of published papers. The M-index assesses the productivity and impact of a researcher by combining the H-index and the number of years since their first publication. It aims to account for the researcher's citation impact and career length [24,25].

In the thematic mapping analysis, clusters representing co-occurring themes are depicted as bubbles on a graph based on Callon's centrality and density rank [26]. Bubble size corresponds to word occurrences within the cluster. The X-axis represents cluster centrality, indicating the level of interaction with other clusters and the significance of a theme. The Y-axis represents density, reflecting the internal strength and growth of a cluster network. The analysis reveals motor themes (high centrality and density), niche themes (high density and low centrality), emerging or declining themes (low centrality and density), and basic themes (high centrality and low density). These findings provide insights into the development and relevance of research themes over time, with upward-right trajectories indicating rising trends and lower-left trajectories indicating declining trends [26,27].

### 2.5 Cross-sectional study

This section involves conducting a national survey among practitioners who utilize CBT in Saudi Arabia. Ethical approval was obtained from the authorized committee at the Deanship of Scientific Research at Jazan University. The questionnaire was randomly distributed to a wide range of participants through email and social networking sites to maximize the sample size. A total of 165 practitioners were included in the sample, which is considered sufficient given the number of practitioners in the country. Specialists in the field were responsible for designing the questionnaire. The questions underwent review by multiple psychologists and linguists to ensure their validity in terms of scientific content, language usage, and suitability for the target sample. The survey is carried out using an electronic questionnaire comprising eleven questions that cover various aspects of CBT, including training, experience, practice, satisfaction, confidence, difficulties, challenges, and outcomes. Questions were closed-ended, utilizing multiple-choice scales. The questionnaire encompassed the following measures: academic specialization and qualifications, the nature of the practitioner's work, years of experience in providing psychological services, sources of training and learning related to CBT, types of psychological disorders treated using CBT, and the extent to which CBT is incorporated in therapy sessions. Additionally, the questionnaire sought the practitioners' opinions on the effectiveness of CBT in improving the health and satisfaction of their clients, the challenges they face when applying CBT within Saudi culture and society, and whether they utilize or recommend any electronic means or applications to support remote CBT. The data was collected using MS-excel sheet and converted to IBM-SPSS for further analysis.

## 3 Results

### 3.1 Scienctimetric findings

#### 3.1.1 Overview and research

The systematic extraction of bibliographic data from Scopus yielded 2757 original studies on CBT for treating PsD. Since its inception in 1979, Fig. 2 demonstrates that this field of study has grown steadily. The polynomial regression coefficient of 0.945 (R^2^) indicates a strong positive trend, and the research has increased at an annual rate of 12.67 %. 12,509 scholars have contributed to this body of research, which is comprised of 864 sources. According to the Scopus indexing of journals, these sources cover a vast array of fields. Research on CBT is significant and multidisciplinary, as evidenced by the many studies and various sources. Medical, psychological, neuroscience, and nursing journals account for approximately 88 % of CBT-related knowledge. This wealth of knowledge provides a solid foundation for future advancements in the field and offers valuable insights into CBT's efficacy and application in treating PsD. The main information for the used data is shown in Table 1.

**Fig. 2:**
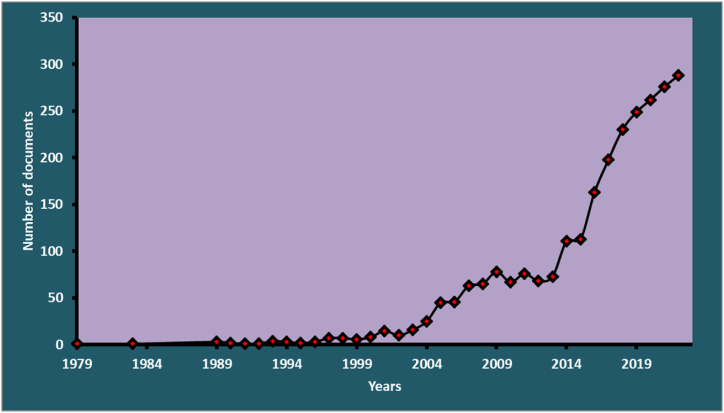
Annual production (1979–2023). Y-axis: the number articles published. X-axis: the years since the first article published in the topic of this paper. Using MeSH-generated keywords and PRISMA guidelines, the Scopus database retrieved bibliographic data (N = 864) without temporal or geographical constraints.

**Table 1:** Main information of the dataset.

Timespan	1979:2023
Sources (Journals)	864
Documents	2757
Annual growth rate %	12.37
Document average age	6.76
Average citations per document	31.45
Keywords plus (ID)	8359
Author's keywords (DE)	4595
Authors	12509
Authors of single-authored documents	186
**Authors collaboration**	
Single-authored documents	197
Co-Authors per documents	5.99
International co-authorships %	22.42

Using MeSH-generated keywords and PRISMA guidelines, the Scopus database retrieved bibliographic data (N = 864) without temporal or geographical constraints.

#### 3.1.2 Hotspots: count and sankey diagram

**Fig. 3:**
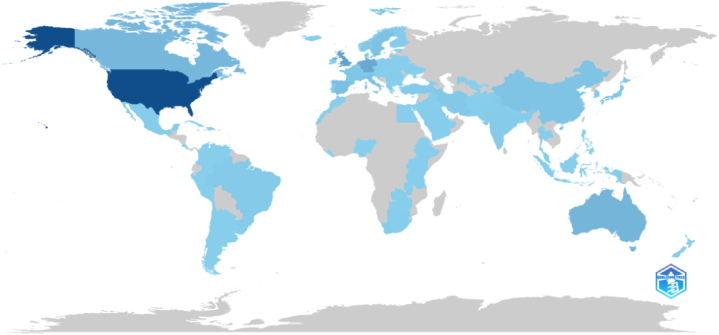
Global research in CBT for the management of PsD. Countries with a dark blue color are the most productive. Countries outside the blue category have not made any contributions to research in this particular area. This figure was generated using the Bibliometrix application and the BibTex data file. Saudi Arabia has 11 documents only. Using MeSH-generated keywords and PRISMA guidelines, the Scopus database retrieved bibliographic data (N = 864) without temporal or geographical constraints. The density of the blue color indicate the number of articles produced by the country. (For interpretation of the references to color in this figure legend, the reader is referred to the Web version of this article.)

Based on the data depicted in Fig. 3, the United States takes the lead as the most productive country, contributing approximately 28 % of the total research output in CBT. The United Kingdom follows with a contribution of 9.81 %, while Germany, Australia, and the Netherlands contribute 8.58 %, 6.31 %, and 5.30 %, respectively. Canada, Italy, Spain, Sweden, and Switzerland also make notable contributions, accounting for 4.69 %, 3.04 %, 2.85 %, 2.56 %, and 2.29 %, respectively. Collectively, these top ten countries account for a significant 75 % of the global research output in CBT, highlighting their predominant role in advancing the field. King's College London (UK), Harvard Medical School (USA), the University of Amsterdam (Netherlands), the Karolinska Institute (Sweden), and Massachusetts General Hospital (USA) are the top publishing institutions. Storch, E.A., affiliated with the University of South Florida, USA, and with 23 documents, is the most prolific author in CBT research. Ranked second and third are Cuijpers, P., and Andersson, G. According to the information, 28 highly productive researchers have produced 10–23 documents. A larger group of 131 researchers has produced 5–10 documents. Note that no subset of researchers produces significantly different results. This suggests that while some researchers are more productive, most produce similar output. It could indicate a balanced distribution of research productivity among researchers without extreme outliers. BMC Psychiatry (74), International Journal of Environmental Research and Public Health (69), Frontiers in Psychiatry (66), Journal of Consulting and Clinical Psychology (61), and Behaviour Research and Therapy (52) are the top journals. These five journals account for 12 % of the CBT research.

**Fig. 4:**
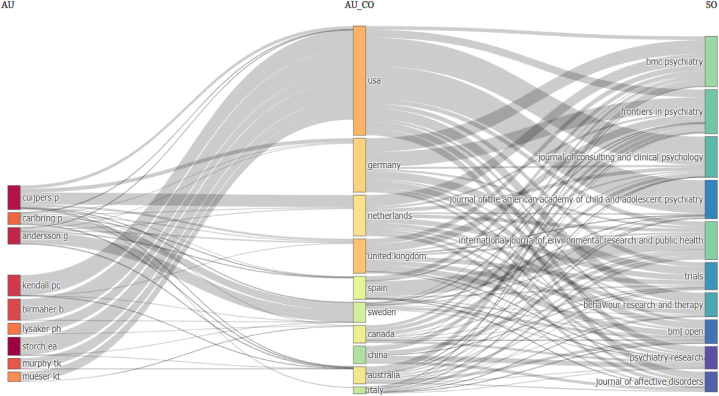
Three field plot. AU: authors; AU_CO: country of the authors; SO: sources. The thickness of the lines connecting authors from different countries would represent the number of papers that they have co-authored. The thickness of the lines connecting countries and sources would represent the number of papers from each country that has been published in each source. Each rectangle represents a node. The size of the rectangle represents the importance of the node in the network. Using MeSH-generated keywords and PRISMA guidelines, the Scopus database retrieved bibliographic data (N = 864) without temporal or geographical constraints. This figure was generated using the Bibliometrix application and the BibTex data file.

Similar to a Sankey diagram, a tripartite diagram is a crucial tool in comprehending the interconnections among variables, such as authorship, source, and country, within the context of bibliometric analysis. Although it does not offer precise measurements, the analysis highlights the comparative associations between the top ten hotspots regarding authors, sources, and countries. The presented visualization facilitates the identification of sources of publication and international research networks. Despite its focus on a subset of the dataset, it provides valuable insights. Fig. 4 shows that Cuijpers, P., is the top publishing author in the most relevant sources. Despite being the researcher with the highest number of publications in the given dataset, Storch, E.A. has now been ranked seventh in the number of research articles published in journals primarily focusing on CBT research. In the same context, the United Kingdom fell to fourth place. Visualizing the relationships would help identify the factors contributing to the UK's decline and reveal any shifts in collaboration patterns, publishing sources, or international research networks that may have contributed to this change in ranking. This would allow for a more comprehensive understanding of the situation and inform strategies for improvement.

#### 3.1.3 Impact of research

In research assessment and knowledge discourse, citation measures a research publication's impact. How often other researchers cite a work indicates its value and contribution to the field. Citation help funders evaluate research quality and influence the scholarly conversation by identifying influential works. The United States is the foremost nation in influential research, boasting a substantial number of citations amounting to 35,940. This figure significantly surpasses the citations of the second-ranked country in the global research landscape, which is five times lower in comparison (Table 2). Indeed, it is notable that no African country appears among the top twenty most influential countries in CBT research, as indicated in Table 2. This observation highlights a potential gap or underrepresentation of African contributions in the field of CBT research. A bibliometric measure known as the average article citations of a country determines the typical number of citations received by articles published by researchers associated with institutions within that nation. It shows the overall impact of the nation's scientific output on research. A higher average article citation score suggests that the articles written in that nation are cited more often, demonstrating a more significant impact and visibility of their research on a global scale. Through our analysis, it is evident that Georgia holds the top position in terms of average article citations (Table 2).

**Table 2:** Top-cited countries.

Rank	Country	Total Citations	Average Article Citations
1	USA	35940	47.40
2	UK	7167	34.10
3	Germany	5670	25.20
4	Netherlands	5370	41.60
5	Australia	4073	29.70
6	Canada	2529	22.20
7	Sweden	1660	23.40
8	Italy	1462	20.00
9	Spain	1119	15.10
10	China	823	14.20
11	Denmark	802	18.20
12	Norway	739	18.90
13	Switzerland	626	20.90
14	Brazil	623	18.30
15	Japan	331	8.30
16	France	316	9.00
17	New Zealand	255	42.50
18	Turkey	225	6.20
19	Georgia	192	64.00
20	Ireland	173	15.70

Using MeSH-generated keywords and PRISMA guidelines, the Scopus database retrieved bibliographic data (N = 864) without temporal or geographical constraints.

Citations are utilized to evaluate the impact of an article, serving as a measure of its influence. However, it is essential to note that the significance of an article cannot be solely determined based on citations. The reason for a newer article having fewer citations is often attributed to its limited readership. Over time, the number of citations for an article typically increases. Table 3 provides a summary of the articles that have been cited. The study by Butler, A. C. received the most citations. This study offers a thorough examination of meta-analyses to evaluate CBT's empirical validity. This study investigates the efficacy of CBT in treating different psychological disorders and provides a comprehensive overview of the current evidence supporting the effectiveness of this therapeutic approach [28].

**Table 3:** Top-cited document.

Rank	Title	Source	Year	Citation	CA
1	The empirical status of cognitive-behavioral therapy: A review of meta-analyses [28]	Clinical Psychology Review	2006	2162	120.11
2	Fluoxetine, cognitive-behavioral therapy, and their combination for adolescents with depression: Treatment for Adolescents with Depression Study (TADS) randomized controlled trial [6]	JAMA	2004	1468	73.40
3	A network theory of mental disorders [30]	World Psychiatry	2017	1210	172.86
4	Management of chronic insomnia disorder in adults: A clinical practice guideline from the American college of physicians [7]	Annals of Internal Medicine	2016	1059	132.38
5	Cognitive behavioral therapy, sertraline, or a combination in childhood anxiety [31]	New England Journal of Medicine	2008	1011	63.19
6	Youth suicide risk and preventive interventions: A review of the past 10 years [32]	Journal of the American Academy of Child and Adolescent Psychiatry	2003	960	45.71
7	A multisite, randomized controlled trial for children with sexual abuse-related PTSD symptoms [33]	Journal of the American Academy of Child and Adolescent Psychiatry	2004	773	38.65
8	Cognitive factors that maintain social anxiety disorder: A comprehensive model and its treatment implications [34]	Cognitive Behaviour Therapy	2007	650	38.24
9	Schizophrenia [9]	Nature Reviews Disease Primers	2015	636	70.67
10	World Federation of Societies of Biological Psychiatry (WFSBP) guidelines for the pharmacological treatment of anxiety, obsessive-compulsive and post-traumatic stress disorders - First revision [35]	World Journal of Biological Psychiatry	2008	580	36.25
11	Transdiagnostic Cognitive-Behavioral therapy for patients with eating disorders: A two-site trial with 60-week follow-up [36]	American Journal of Psychiatry	2009	532	35.47
12	A meta-analysis of the efficacy of acceptance and commitment therapy for clinically relevant mental and physical health problems [30]	Psychotherapy and Psychosomatics	2015	515	57.22
13	Clinical practice guideline for the diagnosis, evaluation, and treatment of attention-deficit/hyperactivity disorder in children and adolescents [8]	Pediatrics	2019	511	102.20
14	Open, aware, and active: Contextual approaches as an emerging trend in the behavioral and cognitive therapies [37]	Annual Review of Clinical Psychology	2011	506	38.92
15	Switching to another SSRI or to venlafaxine with or without cognitive behavioral therapy for adolescents with SSRI-resistant depression: The TORDIA randomized controlled trial [5]	JAMA	2008	483	30.19

Using MeSH-generated keywords and PRISMA guidelines, the Scopus database retrieved bibliographic data (N = 864) without temporal or geographical constraints.

The study by March, J. S. is the second most cited article. This study examines the effectiveness of CBT across various psychological disorders and presents a summary of the existing evidence regarding the efficacy of this therapeutic approach [6]. In order to assess the effectiveness of fluoxetine (an antidepressant), CBT, and their combination in treating depression in adolescents, the Treatment for Adolescents with Depression Study (TADS) conducted a randomized controlled trial. The study's objectives were to evaluate the efficacy of these therapeutic modalities and to shed light on both their individual and combined impacts on adolescent depression. The most successful treatment for easing depressive symptoms in adolescents was found to be the combination of fluoxetine and CBT.

The third-most cited article is a study by D. Borsboom titled "A Network Theory of Mental Disorders." Among the cited articles, it has the highest average number of citations. It was published in the World Psychiatry journal. The study suggests a novel approach to using network theory to comprehend mental disorders. The main conclusions of the study suggest that mental illnesses can be thought of as networks of connected symptoms. The study emphasizes the significance of taking into account the relationships between symptoms and how they influence one another rather than concentrating solely on specific diagnostic categories. This method offers new opportunities for individualized treatment and intervention strategies and sheds light on the dynamic nature of mental disorders [29]. More details are available in Table 3.

**Fig. 5:**
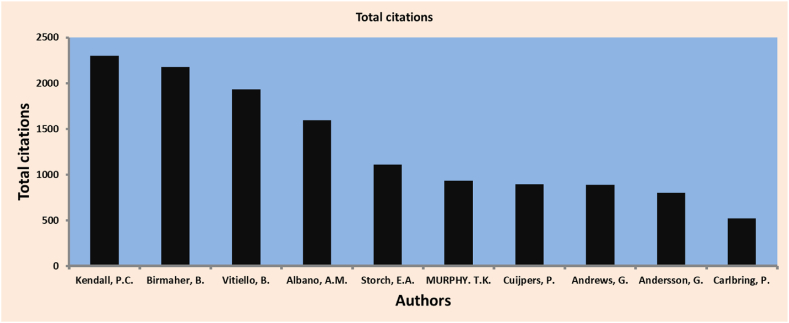
Author local impact by total citation measure.

Local citations refer to the number of times other documents have cited an author or a document within a specific collection that is also included in the same group. In other words, it measures the frequency at which the works within a particular set of documents reference or cite each other. This measure helps assess the interconnectedness and influence of the documents within the collection, providing insights into the scholarly impact of the authors or works contained within that specific context. By examining local citations, researchers can better understand how ideas, research, or theories are referenced and built upon within a specific academic community or field of study. As shown in Table 4 and Fig. 5, the most locally influential scholar in the current bibliographic collection is Kendall, P.C., with an H-index of 15. However, he is the second in terms of H-index. While the author's local impact focuses on the recognition within a specific academic community, the H-index provides a broader assessment of an author's overall impact based on both publication output and citation impact.

**Table 4:** Most-locally impactful researchers.

**Element**	**H_Index**	**G_Index**	**M_Index**	**NP**	**PY_start**
Storch, E.A.	16	23	0.889	23	2006
Kendall, P.C.	15	18	0.441	18	1990
Birmaher, B.	13	15	0.52	15	1999
Vitiello, B.	13	13	0.619	13	2003
Murphy. T.K.	13	15	0.722	15	2006
Cuijpers, P.	13	19	0.765	19	2007
Andersson, G.	13	18	0.591	18	2002
Andrews, G.	11	11	0.579	11	2005
Carlbring, P.	11	13	0.688	13	2008
Albano, A.M.	10	11	0.476	11	2003

Note: NP: number of papers; PY_start: first year of publishing carrier.

#### 3.1.4 Collaborative research

**Fig. 6:**
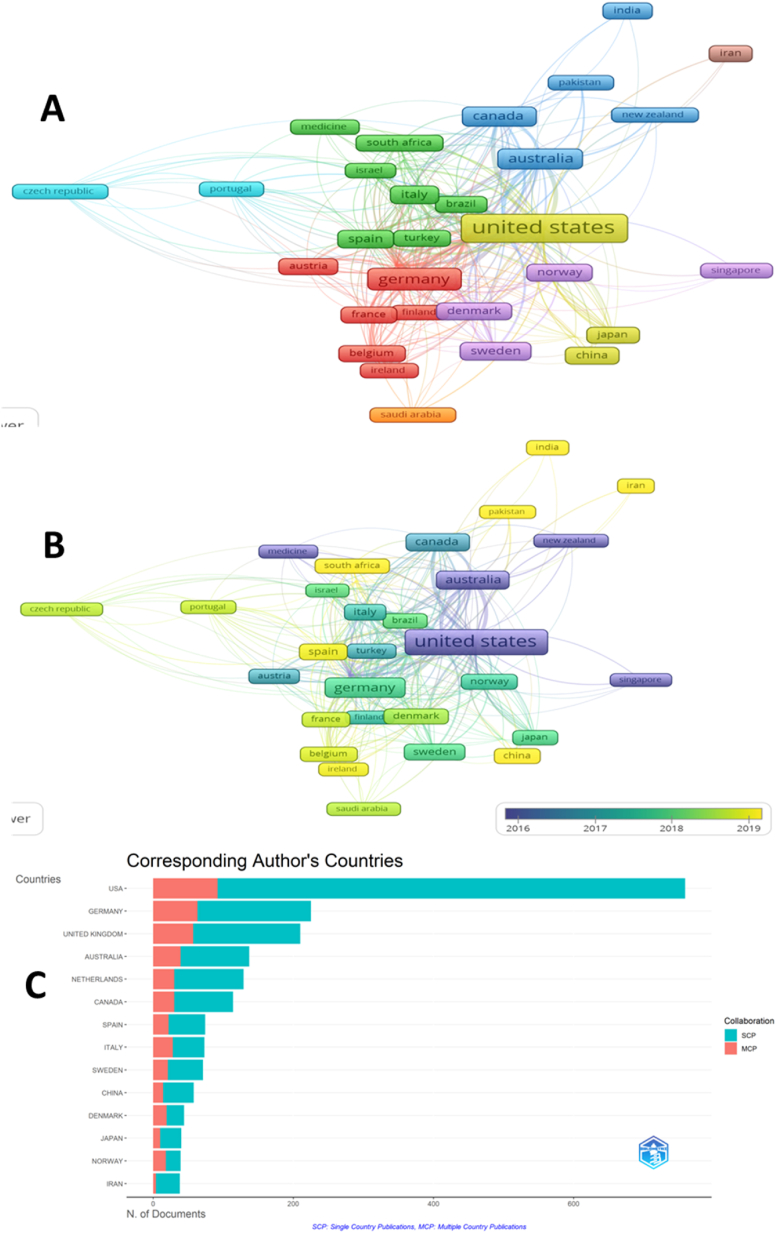
A: Co-authorship networks among countries were analyzed using VOSviewer, where countries were represented as nodes and collaborations as links. Based on TLS values, the United States emerged as the leading collaborative country in CBT research. B: Countries with yellow color were new to the collaborative research in CBT research. Conversely, countries shaded in purple are considered developed and have a long-standing history of research collaboration C: The ratio of national to international collaboration. Interestingly, Germany and the United Kingdom exhibit a higher percentage of international collaboration than the United States. A and B were generated using VOSviewer for countries with a minimum of 10 documents as the mapping threshold. Eight clusters were detected in 6A. Yellow-colored shapes in 6B indicate recent involvement in international collaboration. Bibliometrix was used to generate 6C, which shows the difference between single-country and multiple-country publications. (For interpretation of the references to color in this figure legend, the reader is referred to the Web version of this article.)

Bibliometric maps created with the VOSviewer and Bibliometrix demonstrate how well different countries cooperate in research into CBT as a method of treating PsD. Fig. 6A illustrates the countries active in CBT research into distinct clusters, represented by colors such as yellow, cyan, navy blue, red, violet, brown, and orange. Notably, the United States emerges as the frontrunner, engaging in the highest number of research projects with other nations. Strong collaborations between the United States, Japan, and China indicate their shared commitment to advancing CBT research. The United Kingdom follows closely in second place, while Germany and Australia demonstrate significant participation. On the other hand, Iran and Saudi Arabia form separate clusters, highlighting their independent contributions to CBT research. Some countries, such as China, Ireland, Pakistan, India, and Iran (depicted in yellow in Fig. 6B), are relatively new to international cooperation, seeking to exchange knowledge and experiences with other nations. Yellow-colored shapes in 6B indicate recent involvement in international collaboration. Conversely, countries shaded in purple are considered developed and have a long-standing history of research collaboration.

The number of research projects published within a single nation, as shown in Fig. 6C, sheds light on the ratio of national to international collaboration. Interestingly, Germany and the United Kingdom exhibit a higher percentage of international collaboration than the United States. This suggests that Germany and the United Kingdom actively engage in cooperative efforts with other countries, fostering a global exchange of ideas and expertise in CBT research. Overall, these bibliometric maps serve as valuable tools for evaluating international cooperation and its impact on the effectiveness of research into CBT for PsD. The prominence of certain countries, the formation of distinct clusters, and the varying levels of collaboration provide a comprehensive overview of the global landscape in CBT research, shedding light on successful partnerships and identifying areas for potential growth and knowledge sharing.

#### 3.1.5 Conceptual structure and intellectual evolution

Co-word analysis plays a crucial role in understanding a field's conceptual structure and intellectual evolution. Analyzing co-occurrence patterns of words in scientific literature reveals the relationships between concepts and their evolution over time. It helps identify key concepts, thematic clusters, and emerging trends, providing insights into the intellectual development of a field and facilitating knowledge organization, synthesis, and innovation. Cognitive behavioral therapy (737), depression (317), anxiety (192), mental health (138), treatment (132), psychotherapy (118), children (88), randomized controlled trial (78), schizophrenia (76), adolescents (75), obsessive-compulsive disorder (68), mental disorders (61), insomnia (59), anxiety disorders (54), eating disorders (49), PTSD (48), posttraumatic stress disorder (45), comorbidity (44), psychosis (44), adolescent (43), prevention (43), treatment outcome (43), mental illness (41), and panic disorder (40), are most co-occurred keywords among a total of 4595 author's keywords.

##### 3.1.5.1 Thematic map

**Fig. 7:**
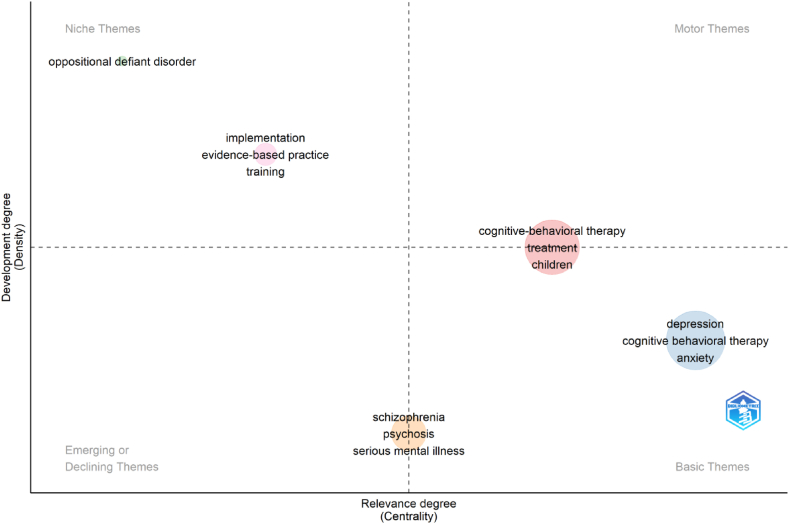
Thematic map of CBT. Thematic maps are divided into four quadrants based on centrality and density, which represent the importance and development of research topics. This figure was generated using the Bibliometrix application and the BibTex data file.

The intellectual landscape of a field is visualized using Bibliometrix software's thematic mapping analysis (Fig. 7). There are four quadrants on the map, which is shown as a scatter plot. The main themes (upper right) are established themes. Emerging themes are a sign of developing research (lower right). Specialized themes (lower-left) signify more focused study areas. Although less central, peripheral themes (upper-left) have substantial publications. Researchers can identify research trends and gain an understanding of the field's structure using this analysis [19,23]. Clusters in the thematic map (Fig. 7) of CBT research are also shown in Table 5. Cognitive-Behavioral Therapy, depression, oppositional defiant disorder, schizophrenia, and implementation are the clusters in the CBT research. Centrality and density decide the localization of each cluster as shown in Table 5 and Fig. 7. The cluster “schizophrenia” with a Callon density of 7.14, ranked first in the density parameter. The building terms for “schizophrenia” cluster are “psychosis,” “serious mental illness,” “severe mental illness,” “recovery,” and “self-esteem” as shown in Table 6. The cluster “depression” is categorized as a basic due to its high value of Callon Centrality. This indicates a high degree of relevance to CBT research. 7.14 is the highest Callon Density, which obtained the niche theme “oppositional defiant disorder”.

**Table 5:** Clusters in the thematic map of CBT research.

Cluster^a^	Callon Centrality	Callon Density	Rank Centrality	Rank Density	Cluster Frequency
Cognitive-Behavioral Therapy	0.44	6.37	4	3	1617
Depression	0.62	6.36	5	2	2588
Oppositional Defiant Disorder	0.00	7.14	1	5	14
Schizophrenia	0.13	5.16	3	1	196
Implementation	0.04	6.54	2	4	52

a In the thematic mapping analysis, clusters representing co-occurring themes are depicted as bubbles on a graph based on Callon's centrality and density rank.

**Table 6:** Terms used to build the thematic map.

Theme	Cluster	Terms	NK
Motor	Cognitive-behavioral therapy	cognitive-behavioral therapy, treatment, children, adolescents, obsessive-compulsive disorder, anxiety disorders, eating disorders, comorbidity, treatment outcome, panic disorder, transdiagnostic, anorexia nervosa, cognitive-behavioural therapy, psychiatric disorders, major depressive disorder, emotion regulation, cognitive behaviour therapy, post-traumatic stress disorder, social anxiety disorder, adhd, bulimia nervosa, generalized anxiety disorder, obesity, assessment, outcome, pharmacotherapy, obsessive compulsive disorder, psychopathology, social phobia, autism spectrum disorder, randomised controlled trial, addiction, adolescence, group therapy, diagnosis, social anxiety, substance use disorder, efficacy, predictors, self-stigma, therapeutic alliance, virtual reality, anxiety disorder, exposure, mood disorders, personality, alcohol dependence, chronic pain, dsm-5, eating disorder, personality disorders	51
Basic	Depression	depression, cognitive behavioral therapy, anxiety, mental health, psychotherapy, cbt, cognitive behavioural therapy, randomized controlled trial, mental disorders, insomnia, ptsd, posttraumatic stress disorder, adolescent, prevention, cognitive behavior therapy, mental illness, internet, intervention, trauma, psychiatry, quality of life, cognitive therapy, covid-19, meta-analysis, primary care, bipolar disorder, effectiveness, stress, mental disorder, mindfulness, common mental disorders, sleep, youth, return to work, acceptance and commitment therapy, child, psychoeducation, rct, mhealth, ocd, telemedicine, veterans, cost-effectiveness, pain, suicide, therapy, adherence, behavior therapy, ehealth, mental health services, motivational interviewing, sick leave, clinical trial, digital health, e-health, exercise, psychosocial interventions	57
Basic to Motor	Oppositional defiant disorder	oppositional defiant disorder	1
Basic to emerging	Schizophrenia	schizophrenia, psychosis, serious mental illness, severe mental illness, recovery, self-esteem	6
Niche	Implementation	implementation, evidence-based practice, training	3

NK: number of keywords; In the thematic mapping analysis, clusters representing co-occurring themes are depicted as bubbles on a graph based on Callon's centrality and density rank.

##### 3.1.5.2 Thematic evolution

**Fig. 8:**
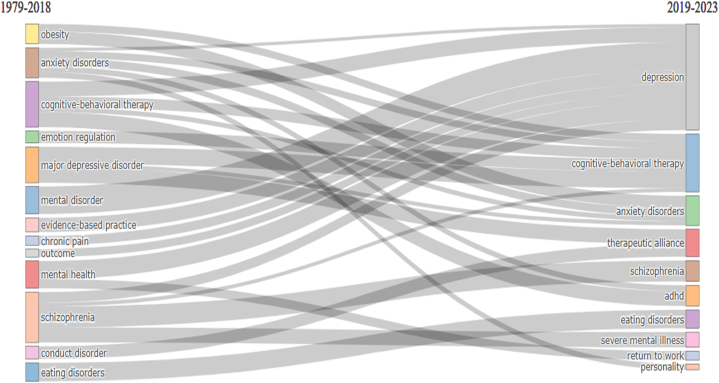
Thematic evolution. 2018 was a crucial point for the transformation of the main topics. This figure was generated using the Bibliometrix application and the BibTex data file.

**Fig. 9:**
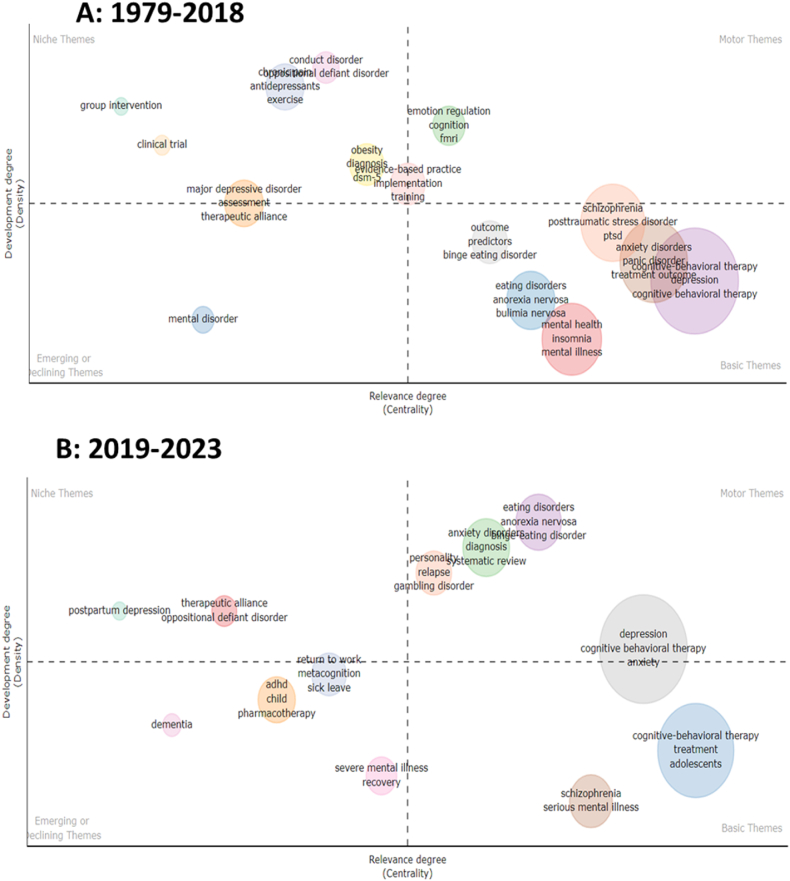
Mapping of the tow period of the thematic evolution.

Fig. 8 depicts the chronological progression of CBT in terms of its thematic development, spanning from its initial conceptualization to the present year. The year 2019 marked a significant milestone in advancing CBT research. During the pre-2019 period, 13 distinct research clusters were identified. These clusters encompassed a range of topics, including anxiety disorders, chronic pain, cognitive-behavioral therapy, conduct disorder, eating disorders, emotion regulation, evidence-based practice, major depressive disorder, mental disorder, schizophrenia, mental health, obesity, and outcome. However, it is noteworthy that the number of research clusters decreased to 10 after 2019. Of the various clusters examined, only four demonstrated concurrence across both periods. These clusters were identified explicitly as "obesity," "CBT," "schizophrenia," and "eating disorders." Indeed, subsequent to the year 2019, a total of six novel clusters have surfaced, namely "depression," "therapeutic alliance," "ADHD," "severe mental illness," "return to work," and "personality." The field of obesity research has experienced a decline in prominence and has become integrated within the broader domains of cognitive-behavioral therapy (CBT) and anxiety disorders. The emergence of the depression cluster after 2019 can be attributed to the amalgamation of eight distinct clusters that existed prior to the aforementioned year. Fig. 9A and B shows that certain clusters have undergone spatial displacement within the conceptual map, suggesting the progression of intellectual inquiry in the field of CBT. As an illustrative instance, it is worth mentioning that the cluster associated with "anxiety disorder" has transitioned from being a peripheral aspect to assuming a central role. This observation pertains to the cluster of eating disorders. It is also observed that the cluster labeled "separation" exhibits a relatively lower density value along the vertical axis and is evidently situated within the overarching theme.

##### 3.1.5.3 Trending topics

**Fig. 10:**
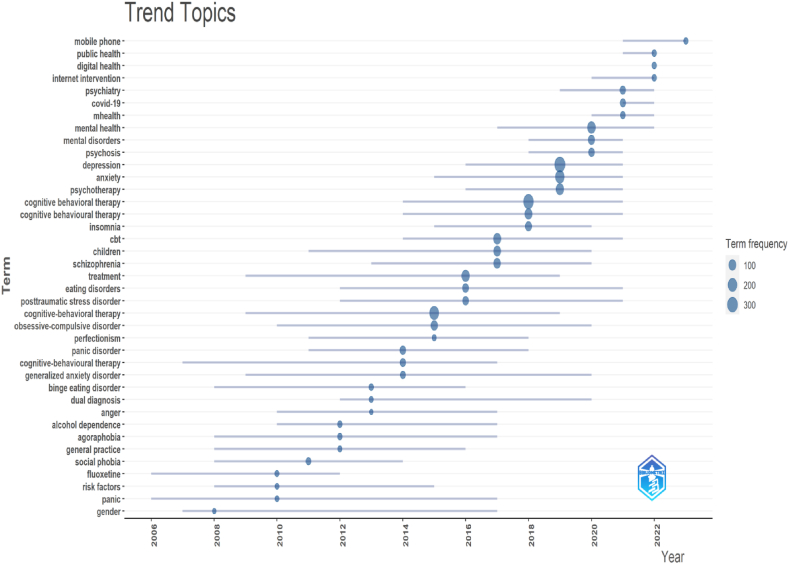
Trending topics. The graph depicts the research topic's time span, with horizontal lines indicating the duration and blue circles representing the frequency of the term. This figure was generated using Bibliometrix and BibTex data files. (For interpretation of the references to color in this figure legend, the reader is referred to the Web version of this article.)

Trending topics in a field are analyzed using the Bibliometrix application. First, pertinent bibliographic information, including article titles, abstracts, and keywords, is gathered. The software then uses sophisticated algorithms to find patterns and connections in the data. It can reveal well-liked research topics, new topics, and significant authors or publications. Bibliometrix creates visualizations like network graphs to show the distribution and relationships among topics. Trending topics in CBT are shown in Fig. 10. The bibliometric analysis underscores the nascent research patterns that establish connections between mobile phones, mHealth, internet interventions, and digital health within the framework of COVID-19 and public health (Fig. 10). This study illuminates the effectiveness of CBT as a valuable therapeutic intervention for PsD, employing innovative technological methodologies. Numerous research areas, such as generalized anxiety disorder, panic disorder, eating disorders, and posttraumatic stress disorder, have consistently maintained their significance in CBT research. The horizontal line depicted in Fig. 10 serves as a visual representation of the temporal extent of the research cluster. On the contrary, the circles in the diagram represent the degree of strength, with the cluster of depressions being the most widespread.

### 3.2 Cross-sectional investigations

The section offers a comprehensive overview of the background of the study's respondents. It presents information on their professional specialization, academic qualifications, job settings, years of experience, and sources of training related to CBT (Table 7). The majority of respondents (81.1 %) specialize in Psychology, while others come from diverse fields such as Social Service, Kindergarten, Psychiatry, Sociology, and Special Education. In terms of academic degrees, 42.7 % hold a Bachelor's degree, 43.9 % have a Master's degree, and 13.4 % possess a Doctoral degree. The respondents work in various job settings, with 64.0 % employed in health institutions, 5.5 % in the community, 17.7 % in schools or educational institutions, and 12.8 % in rehabilitation and special care centers. The data also provides insights into the respondents' years of experience, ranging from less than one year to over 15 years. Furthermore, the table reveals the sources of training for the respondents, including courses, workshops, conferences, articles, institutional training, books, videos, and other avenues. By offering this background information, the table enhances our understanding of the demographics and characteristics of the respondents, enabling a contextualized exploration of their perspectives and experiences concerning CBT and psychological disorders in Saudi Arabia.

**Table 7:** Background of the respondents (N = 164).

**Variables**	N	%
**Specialty**		
Psychology	133	81.1
Kindergarten	1	0.6
Social service	16	9.8
Psychiatry	10	6.1
Sociology	3	1.8
Special Education	1	0.6
**Academic degree**		
BSc	70	42.7
MSc	72	43.9
PhD	22	13.4
**Job**		
Working in health institutions (such as hospitals, clinics, or health centers)	105	64.0
Work in the community (such as volunteer work, social programs, or psychological and family counseling centers)	9	5.5
Working in schools or educational institutions	29	17.7
Working in rehabilitation and special care centers (such as end of-life care, nursing home, comprehensive rehabilitation)	21	12.8
**Experience**		
Less than one year	22	13.4
1–5 years	45	27.4
6–10 years	37	22.6
11–15 years	29	17.7
More than 15 years	31	18.9
**Source of training (Yes)**		
Books, articles or videos	67	40.9
Courses, workshops or conferences	118	72.0
Supervision or consultation from an expert	15	31.1
Specialized institutional training with supervision	37	22.6
Others	11	6.7
Use of CBT		
Always	35	21.3
Mostly	60	36.6
Sometimes	50	30.5
Scarcely	12	7.3
I don't use it at all	7	4.3
**Perception of the respondents on the efficiency of CBT**		
Inefficient	3	1.8
Average effectiveness	47	28.7
Effective	85	51.8
Very effective	29	17.7
**Do you use or recommend any electronic methods or applications to support CBT remotely?**		
Yes	57	34.8
No	107	65.2

An extensive synopsis of the respondents' backgrounds to the study is provided in this table. It provides details on their years of experience, employment settings, academic background, area of expertise, and CBT training sources. A total of 165 practitioners were included in the sample, which is considered sufficient given the number of practitioners in the country. Specialists in the field were responsible for designing the questionnaire.

The provided table reveals the frequency of CBT utilization among the respondents. Among the participants, 35 individuals (21.3 %) reported always employing CBT in their practice, while 60 respondents (36.6 %) stated that they mostly use CBT [38]. Additionally, 50 respondents (30.5 %) reported using CBT occasionally, whereas 12 participants (7.3 %) indicated scarce utilization of CBT. A small portion of the respondents, comprising 7 individuals (4.3 %), stated that they do not use CBT at all. These findings provide valuable insights into the usage patterns of CBT among the practitioners, highlighting a significant proportion who consistently or frequently incorporate CBT into their therapeutic approaches. However, a portion of the respondents appears to have limited or no reliance on CBT. Understanding such patterns of utilization can contribute to a better comprehension of the implementation and acceptance of CBT within the context of psychological disorders in the Kingdom of Saudi Arabia.

The respondents' perceptions regarding the efficiency of CBT varied. A small percentage (1.8 %) considered CBT to be inefficient, while a significant portion (51.8 %) perceived it as effective. Additionally, 28.7 % viewed CBT as having an average level of effectiveness, and 17.7 % regarded it as very effective. These findings highlight that the majority of respondents held positive perceptions of CBT, considering it either effective or highly effective in addressing psychological disorders. However, it's important to acknowledge that a minority of respondents expressed a perception of inefficiency. Understanding these varied perceptions provides valuable insights into the attitudes and beliefs of practitioners towards the effectiveness of CBT in the context of psychological treatment.

**Fig. 11:**
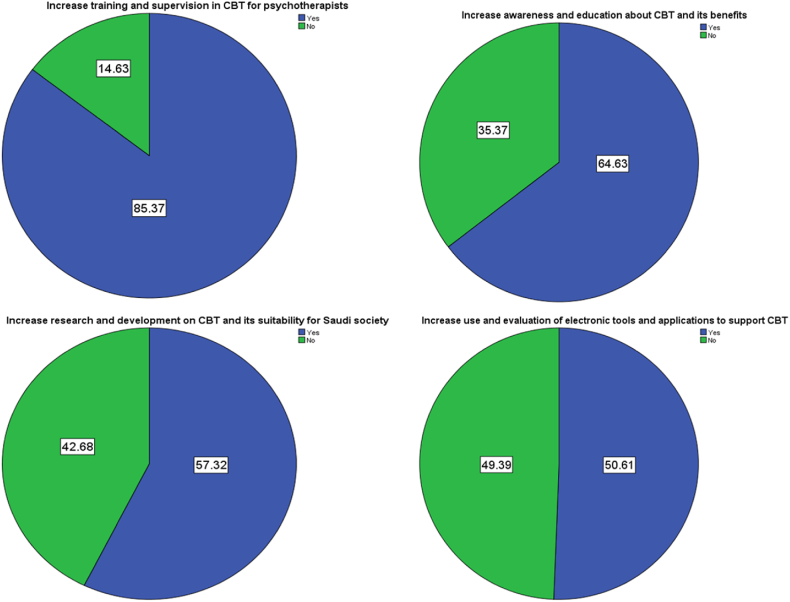
Suggestions and Recommendations for Developing and Disseminating CBT in the Kingdom of Saudi Arabia. A total of 165 practitioners were included in the sample, which is considered sufficient given the number of practitioners in the country. Specialists in the field were responsible for designing the questionnaire.

According to the respondents, several suggestions and recommendations emerged for the development and dissemination of CBT in Saudi Arabia (Fig. 11). The majority (85.4 %) emphasized the importance of increasing training and supervision opportunities in CBT for psychotherapists, indicating a need to enhance their knowledge and skills in CBT techniques. Additionally, a significant proportion (64.6 %) highlighted the necessity of raising awareness and education about CBT and its benefits among professionals and the general public. Respondents also emphasized the need for increased research and development efforts (57.3 %) to explore the suitability of CBT for Saudi society and to tailor approaches to the local context. Furthermore, a notable percentage (50.6 %) suggested utilizing and evaluating electronic tools and applications to support CBT, recognizing the potential benefits of technology in improving accessibility and delivery of CBT interventions. These recommendations collectively underscore the importance of investing in training, awareness, research, and technology to advance the development and dissemination of CBT within the mental health landscape of Saudi Arabia.

## 4 Discussion

This study offers practical contributions that can benefit researchers, practitioners, policymakers, and healthcare professionals in the field of CBT. It identifies key research trends, influential studies, and research gaps, helping guide future research efforts and directing attention towards important areas of investigation. By highlighting the contributions of different countries and examining the impact of COVID-19 on internet-based interventions, the study provides a global perspective and informs collaborative efforts for the dissemination and implementation of effective CBT interventions. The contextual insights specific to Saudi Arabia shed light on the development and dissemination of CBT in that region, offering guidance for tailored strategies in training, awareness, and research. Overall, these practical contributions enhance the understanding of CBT research trends, inform evidence-based practices, and support the advancement of CBT interventions for improved mental health outcomes.

### 4.1 Bibliometric study

The present study emphasized its significance while observing greater dynamism in research, particularly in applying CBT to PsD. CBT has emerged as a crucial aspect of research across various domains, highlighting a noteworthy surge over the past two decades, commencing in 2004. The current findings are in line with previous research which indicated the growth of CBT research [1,39]. Understanding research output distribution can help assess community productivity and identify areas needing additional support or resources [40]. Further data analysis suggests a balanced distribution of research productivity among the 131 researchers, with most producing a similar output of 5–10 documents. While some researchers may be more productive, there are no significant differences within subsets of researchers. However, it is worth noting that no African country appears among the top twenty most influential countries in CBT research, indicating a potential gap or underrepresentation of African contributions in the field. Addressing this disparity is important to promote inclusivity and diversity in CBT research by focusing on areas such as research funding, collaboration opportunities, and capacity-building initiatives in African countries. The current study is consistent with previous research regarding the scarcity of research on CBT in Africa [3,41].

The COVID-19 pandemic has rendered this crucial subject pertinent in contemporary society. COVID-19 has significantly impacted the adoption and efficacy of internet-based interventions for mental health [[42], [43], [44], [45], [46]]. Due to limitations on in-person services, there has been an increase in the use of online platforms, which has led to a broader adoption of internet interventions [46,47]. For those who face isolation and have limited access to traditional services, these interventions have offered convenient and easily accessible mental health support. However, obstacles like the digital divide and a lack of technological literacy have made it harder for everyone to access these interventions equally. The effectiveness of internet interventions has also been the subject of ongoing research, with results pointing to successful outcomes in symptom reduction and well-being improvement for some mental health conditions. Individual characteristics, such as motivation and interest, can, however, affect how effective they are overall. Despite these difficulties, the pandemic has sped up technology adoption in mental health care and highlighted the potential of online interventions to enhance access to mental health care. Research gaps in CBT include understanding the underlying mechanisms of change, individual differences in treatment response, long-term outcomes, treatment personalization, dissemination challenges, technology-based interventions, and cultural adaptation for diverse populations.

The thematic evolution analysis of cognitive-behavioral therapy (CBT) research provides valuable insights for future research directions and clinical practice in CBT for psychological disorders (PsD). The identification of enduring research clusters, such as "obesity," "CBT," "schizophrenia," and "eating disorders," highlights areas of consistent focus. Additionally, the emergence of novel clusters like "depression," "therapeutic alliance," and "ADHD" indicates expanding domains for CBT research. The integration of obesity research within broader domains [48], the consolidation of knowledge in the depression cluster [1,5,49], and the shifts in the conceptual map, particularly with anxiety disorders and eating disorders, emphasize the interconnectedness and evolving understanding of PsD [50]. While the use of antidepressant medication is controversial due to concerns about efficacy and safety, CBT has shown promise as an alternative treatment option. CBT helps patients regulate their emotions, engage in meaningful activities, and maintain realistic thinking. These findings inform researchers and clinicians to prioritize key areas, explore new domains, address comorbidity, and employ integrated approaches to enhance CBT's efficacy in treating psychological disorders.

#### 4.1.1 Limitations

There are a number of limitations to bibliometric research. It is predominately quantitative and disregards qualitative aspects of research. Self-citation and publication bias are examples of data limitations and biases that can distort results. Lack of contextual information and inability to document emerging research can limit the exhaustiveness of a work. Bibliographic analysis is limited by incomplete database coverage and the difficulty of accurately depicting research impact and significance. The prevalence of English-language publications in bibliometric databases can introduce language bias. Furthermore, there may be disparities in the representation of research from various geographical regions, especially non-English-speaking or developing nations.

### 4.2 Cross-sectional study

The bibliometric analysis revealed that Saudi Arabia's contribution in terms of performance, cooperation, and impact is considerably lower compared to top-producing countries. This finding prompted our research team to conduct a cross-sectional study to analyze the current state of CBT in Saudi Arabia.

The background information of the study's respondents offers valuable insights into their professional qualifications, experiences, and sources of training related to CBT in Saudi Arabia. The respondents represent a range of professional specializations, including Psychology, Kindergarten, Social Service, Psychiatry, Sociology, and Special Education. They hold various academic degrees, such as Bachelor's, Master's, and Doctoral degrees. The respondents work in diverse job settings, including health institutions, community settings, schools or educational institutions, and rehabilitation and special care centers. They have varying years of experience in their respective fields. The respondents have sought training from multiple sources, such as books, articles, videos, courses, workshops, conferences, supervision, institutional training, and other avenues. This comprehensive background information provides a contextual understanding of the respondents' qualifications and experiences, which informs their perspectives, suggestions, and recommendations for the development and dissemination of CBT in Saudi Arabia. Globally, CBT is practiced by a diverse group of mental health professionals who have received specialized training in this approach. Their expertise and skills in CBT enable them to provide effective and evidence-based treatment for individuals experiencing various mental health challenges [38].

The results indicate that among the respondents in the study, a relatively lower frequency of CBT utilization was reported in the context of Saudi Arabia. Approximately 21.3 % of participants stated that they always employ CBT in their practice, while 36.6 % reported mostly using CBT. Several factors may contribute to the comparatively lower utilization of CBT in KSA. Cultural factors, such as the influence of cultural norms and beliefs prioritizing alternative treatment modalities, could play a role [51]. Limited awareness and familiarity with CBT among mental health professionals, potentially due to limited training and education opportunities specific to CBT, might also contribute [52]. Treatment culture and preferences, both among professionals and the general population, as well as the availability and dissemination of research supporting the efficacy of CBT in the Saudi Arabian context, could influence its utilization [53]. Further research is needed to gain a deeper understanding of these factors and their impact on the utilization of CBT in KSA.

The study found that 34.8 % of respondents reported using or recommending electronic methods or applications to support CBT remotely, while 65.2 % answered that they do not utilize or recommend such methods. The use of electronic methods and applications in remote CBT offers several potential benefits, including enhanced accessibility, convenience, and the ability to provide virtual self-help resources and tools for practicing coping skills [54]. However, there are also concerns about the effectiveness and security of electronic platforms, and the decision to use them may depend on the specific context, resources, and regulations in the region where therapists practice [55]. As technology continues to advance, it is likely that the utilization of electronic methods and applications in CBT will continue to evolve [56]. Further research is needed to guide therapists in effectively integrating these tools into remote CBT practice.

The majority of respondents in this study emphasized the need to increase training and supervision opportunities in Cognitive Behavioral Therapy (CBT) for psychotherapists. This recommendation reflects recognition of the importance of enhancing the knowledge and skills of mental health professionals in CBT techniques. By providing comprehensive training and supervision, professionals can improve their competency in delivering CBT interventions, leading to more effective treatment outcomes and the overall improvement of mental health services in KSA. Ongoing professional development and staying abreast of advancements in the field are also recognized as crucial. Investing in the development and dissemination of CBT through increased training and supervision can contribute to improved mental health care in KSA. Branson et al., emphasized on the role of training to increase CBT competence in the USA [49]. Patients who received treatment from therapists with lower levels of competence consistently exhibited greater deterioration in their symptoms compared to what would be typically anticipated [57].

The limited utilization of cognitive-behavioral therapy (CBT) in Saudi Arabia compared to other countries can be attributed to various cultural, societal, and healthcare system factors. Saudi Arabia's conservative cultural context may influence the acceptance and utilization of CBT, with traditional beliefs and norms potentially contributing to stigma and reluctance in seeking psychological interventions [58]. Limited awareness and education about CBT among the general public and healthcare professionals, as well as a shortage of trained practitioners, further hinder its utilization [59]. Additionally, the availability of CBT services, language and cultural adaptation of interventions, and healthcare system factors such as resource limitations and lack of integration of mental health services into primary care settings impact CBT's accessibility [60]. Addressing these factors requires raising awareness, promoting mental health literacy, providing comprehensive education and training, establishing specialized CBT services, adapting interventions to the local context, and integrating mental health into primary healthcare. Collaboration between international experts and local researchers can contribute to the development and dissemination of culturally appropriate CBT practices in Saudi Arabia.

#### 4.2.1 Limitations

The cross-sectional study design has limitations, including selection bias and reliance on self-reported data. Future studies should aim for diverse samples and incorporate objective measures to mitigate these limitations. Longitudinal and mixed-methods approaches can provide comprehensive insights. Comparative studies across regions and collaborative research efforts are important for understanding CBT utilization. These strategies enhance generalizability and inform culturally sensitive approaches in Saudi Arabia.

## 5 Implications and future studies

The study evaluated CBT research in treating PsD, which can provide valuable recommendations to guide future research efforts. The study identified underrepresented PsD, specific populations, or treatment modalities that have received limited attention in CBT research. Recommendations include directing research efforts toward filling these gaps to ensure a more comprehensive understanding of CBT's efficacy across diverse disorders and populations. Bibliometric analysis can reveal productive research teams and institutions. Interdisciplinary collaborations are encouraged between researchers from psychology, psychiatry, neuroscience, and other relevant fields. Such collaborations can lead to enriched methodologies, innovative interventions, and a more comprehensive understanding of CBT's mechanisms of change. Additionally, the study recommends using rigorous research designs, such as randomized controlled trials, to improve the quality of future studies. Using such designs, researchers can ensure a more rigorous evaluation of CBT interventions, leading to more reliable and generalizable findings. These recommendations aim to strengthen the scientific foundation of CBT and provide a solid basis for evidence-based practice in treating PsD. The study revealed emerging trends in using digital platforms, mobile applications, virtual reality, or wearable devices within CBT interventions. Recommendations may involve exploring the integration of these technologies to enhance treatment outcomes. Further research can evaluate the efficacy, accessibility, and cost-effectiveness of these technology-based interventions in the context of CBT. By leveraging technological advancements, researchers can potentially improve the delivery, accessibility, and effectiveness of CBT, expanding its reach and impact in the treatment of PsD. Furthermore, this study highlighted the importance of cultural adaptation in CBT research. Recommendations may include cultural adaptation to make CBT interventions relevant and effective across diverse populations. Future research can adapt CBT techniques, treatment manuals, and therapist training to align with the values, thoughts, and practices of different ethnic and cultural groups. Moreover, the study suggests the need for more research on the long-term outcomes of CBT interventions. Follow-up studies may be recommended to assess the longevity of treatment effects. This can provide insights into the long-term benefits of CBT and inform strategies for relapse prevention and sustained well-being. By implementing these recommendations, researchers can improve the quality, relevance, and impact of CBT research for PsD. This, in turn, can lead to improved treatment outcomes, contribute to the field's knowledge base, and advance evidence-based mental health practice.

Based on the cross-study, several recommendations and conclusions can be drawn. Firstly, there is a need to increase training and supervision opportunities in CBT for mental health professionals in Saudi Arabia. Enhancing their knowledge and skills in CBT techniques can lead to improved treatment outcomes and the delivery of high-quality care. Additionally, promoting awareness and education about CBT among professionals and conducting further research to understand its effectiveness in the local context are crucial steps in developing and disseminating CBT in Saudi Arabia.

## 6 Conclusion

By following this method, a systematic and comprehensive evaluation of CBT research related to mental illnesses was conducted, utilizing Scopus as the primary database. The exclusion of non-English language research articles ensured consistency in the dataset, while the PRISMA guidelines facilitated a rigorous and transparent approach to data extraction. A bibliometric analysis of research on CBT provides a thorough and quantitative assessment of the field's advancements, contributions, and influence. This study demonstrates that the prominence of cognitive behavioral therapy research has grown significantly in the last three to four decades, garnering extensive recognition within the mental health domain. The obtained results provide a heightened sense of reassurance, as evidenced by the data presented. The data also indicate a relatively even distribution of cooperation. Nevertheless, there remains a potential for further development in this area, particularly within emerging and industrialized economies. This study aims to establish a comprehensive research framework to address a topic relevant to countries that have yet to embrace this treatment approach. The research area under consideration exhibits promising prospects for expansion, primarily attributable to the emergence of subcategories and an enhanced diversity of keywords within the CBT field. The extensive scope and concentration of keywords indicate the considerable diversity of the subject matter. The findings will have implications for improving treatment approaches, developing evidence-based guidelines, and guiding future research efforts in the field of CBT for psychological disorders.

To promote the development and dissemination of cognitive-behavioral therapy (CBT) in Saudi Arabia, policymakers and healthcare organizations should establish comprehensive training programs for healthcare professionals, allocate resources for CBT research, and collaborate with international experts. They should integrate CBT into mental health services, develop culturally adapted interventions, increase public awareness, establish specialized CBT clinics, and implement telehealth and digital platforms. These actions will enhance the knowledge and skills of professionals, facilitate research, ensure cultural appropriateness, improve access to CBT services, and raise awareness about the benefits of CBT in Saudi Arabia.

## Institutional review board statement

Not applicable.

## Informed consent statement

Not applicable.

## Data availability statement

All data associated with this study will be available based on the reasonable request to corresponding author.

## CRediT authorship contribution statement

**Hadi Dhafer Hassan Kariri:** Writing – review & editing, Writing – original draft, Visualization, Validation, Supervision, Software, Resources, Project administration, Methodology, Investigation, Funding acquisition, Formal analysis, Data curation, Conceptualization. **Abdulmohsen Almubaddel:** Writing – review & editing, Writing – original draft, Visualization, Validation, Supervision, Software, Resources, Project administration, Methodology, Investigation, Funding acquisition, Formal analysis, Data curation, Conceptualization.

## Declaration of competing interest

The authors declare that they have no known competing financial interests or personal relationships that could have appeared to influence the work reported in this paper.
